# Mortality Related to Different Circulatory Diseases Before, During and After the COVID‐19 Pandemic

**DOI:** 10.1002/clc.70433

**Published:** 2026-07-31

**Authors:** Giulia Capodaglio, Ugo Fedeli, Susanna Levantesi, Paolo Girardi

**Affiliations:** ^1^ Screening Unit, Azienda Zero Veneto Region Padova Italy; ^2^ Epidemiological Department Azienda Zero, Veneto Region Padova Italy; ^3^ Department of Statistics Sapienza University of Rome Roma Italy; ^4^ Department of Environmental Sciences Informatics and Statistics, Ca' Foscari University of Venice, Mestre‐Venezia Italy

**Keywords:** circulatory diseases, COVID‐19, mortality, time series analysis

## Abstract

**Background:**

The COVID‐19 pandemic caused a global rise in cardiovascular mortality. In Italy, a large excess deaths from ischemic heart disease (IHD), cerebrovascular disease (CVD), hypertensive diseases (HD), and atrial fibrillation (AF) was observed early during the pandemic. Whether mortality has since returned to pre‐pandemic levels remains uncertain.

**Methods:**

This study analyzed mortality data for residents aged ≥ 40 in the Veneto Region, Italy, using multiple causes of death (MCOD) from death certificates. Age‐standardized monthly mortality rates were computed for IHD, CVD, HD, and AF. Forecasts for 2023 were generated using two scenarios: excluding the pandemic period (2008–2019) and including it (2008–2022). Seasonal ARIMA and Lee‐Carter models were applied, and observed 2023 rates were compared with forecasts.

**Results:**

In 2020, all circulatory disease categories showed a mortality peak, especially HD (+29%). Although rates decreased in 2021–2022, they remained significantly above pre‐pandemic levels. In 2023, observed rates for all conditions were close to those forecasted using the 2008–2019 baseline. By contrast, observed rates were significantly lower than expected when using the 2008–2022 baseline. Seasonal peaks were still observed in December 2023, associated with the circulation of multiple respiratory viruses. Lee‐Carter model results confirmed a general re‐alignment of 2023 mortality with pre‐pandemic patterns.

**Conclusions:**

By 2023, mortality from major circulatory diseases in the Veneto Region largely returned to pre‐pandemic trends, suggesting limited long‐term excess mortality. The findings underscore the value of MCOD data and time‐series modeling in capturing both short‐term disruptions and long‐term recovery patterns in population health.

## Graphical abstract

The COVID‐19 pandemic triggered significant mortality spikes for circulatory diseases (IHD, CVD, HD, and AF) in Veneto between 2020 and 2022. However, by 2023, mortality rates for all conditions realigned with pre‐pandemic historical trends, indicating that the long‐term impact on cardiovascular mortality remained limited.

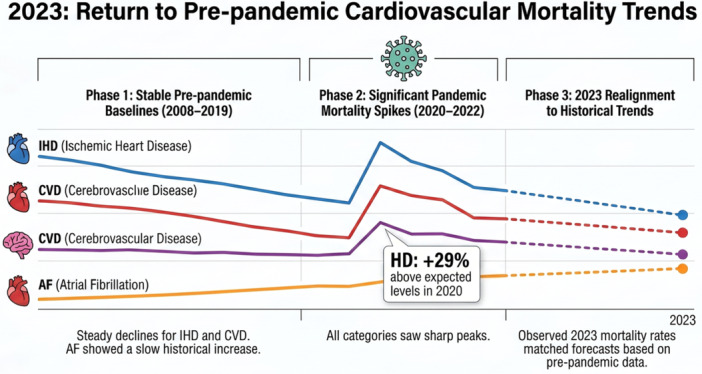

## 1 Introduction

The CoronaVirus Disease 2019 (COVID‐19) pandemic had a profound impact on cardiovascular disease mortality among the general population, with significant increases observed globally due to several factors, which cover COVID‐19 complications, delayed medical care, and increased stress related due to pandemic conditions [1]. Observational data showed increased mortality in the year 2020 from ischemic heart diseases (IHD) and cerebrovascular diseases (CVD) in several countries [2]. An association between peaks in mortality from cardiovascular diseases and COVID‐19 incidence was found [3]. However, most reports relied on routine mortality statistics limited to the underlying cause of death (UCOD), selected from all conditions reported on death certificates based on international coding rules. Analyses extended to all diseases mentioned in certificates (multiple causes of death‐MCOD) allowed to assess larger increases in mortality related to IHD, CVD, hypertensive diseases (HD) and atrial fibrillation (AF) during the first phase of the pandemic in Italy [4].

It remains to be assessed if mortality related to circulatory diseases returned to pre‐pandemic trends as health care systems recovered and vaccination efforts advanced. In fact, mortality might have further decreased after the end of the pandemic if peaks registered were mostly due to an acceleration in the process of death among extremely vulnerable individuals (i.e., mortality displacement), or by the contrary might have remained above previous trends if the pandemic had a longer term impact due to direct (long‐COVID) and indirect (diagnostic and therapeutic delays) effects [5]. Detailed analyses of causes of mortality after the end of the pandemic are mainly available from the US. Overall age‐standardized mortality in 2023 remained above pre‐pandemic levels, but patterns by cause of death were markedly different [6]. Cardiovascular mortality as a whole dropped in the US in 2023 after peaks registered in 2020–2022, but remained higher than values predicted on the basis of pre‐pandemic trends [7]. Namely, in 2022–2023 IHD as the UCOD declined below figures registered in 2018–2019, whereas an increase was still observed for CVD [8], HD [9, 10], and AF [11]; such findings seem to be confirmed by 2024 provisional data [12]. Sparse data are available from Europe. In the UK, an excess in mortality from all cardiovascular diseases and from IHD was still registered between June 2022 and June 2023 [13]. Different patterns could be observed by age group [14]; despite a decrease between 2023 and 2024, the male all‐cause mortality rate under age 75 in England remained higher than in 2019 [15]. In France, while decreasing in 2023, excess mortality remained “substantial” for the fourth consecutive year [16]. Lastly, previous analyses carried out in Northern Italy showed that IHD‐related mortality based on MCOD peaked in 2020; in 2021–2022 mortality decreased towards rates observed in 2018–2019 [17].

The present study aims at filling the knowledge gap on post‐pandemic cardiovascular mortality trends in Europe. To this purpose, we analyzed mortality rates related to different circulatory diseases (IHD, CVD, HD, and AF) to assess the potential return to pre‐pandemic trends in 2023 in the Veneto Region (Italy), one of the first and most severely hit European areas by the pandemic in early 2020 [4]. To this purpose, multiple analytic approaches applied to the 2008–2023 mortality time series based on MCOD were adopted.

## 2 Methods

### 2.1 Data Source

Mortality data by cause of death were derived from death certificates of residents in the Veneto Region (northeastern Italy), which in 2023 had a population of approximately 4.9 million. A copy of all certificates is collected by the Regional Epidemiological Department, which also takes care of coding in accordance with the International Classification of Diseases, 10th revision (ICD‐10). Starting from 2008, all conditions reported in death certificates are coded and included in the MCOD archive. All death certificates from January 1, 2008 until December 31, 2023 with any mention of IHD (ICD‐10 codes I20‐I25), CVD (I60‐I69), AF (I48) or HD (I10‐I15) were retrieved from the regional mortality registry, in accordance with prior research [4]. The yearly and monthly number of deaths related to IHD, CVD, HD or AF were calculated for 5‐year age groups starting from 40 years old. Age‐standardized mortality rates were computed based on the 2013 European standard population to minimize the effects of changes in the population age structure.

The study was based on anonymized data; the analysis of mortality records is a mandatory activity of the Regional Epidemiological Department according to the regional law. Therefore, this study was exempt from Institutional Review Board approval.

### 2.2 Statistical Analysis

To assess the potential return to pre‐pandemic trends in 2023, we considered the historical series of mortality data, considering two forecasting scenarios for the year 2023. The first scenario excluded the pandemic period, using data from 2008 to 2019 for the fitting process, while the second was based on the complete historical series from 2008 to 2022.

Our main mortality modeling approach of age‐standardized monthly mortality rates was based on Autoregressive Integrated Moving Average (ARIMA) models in order to capture temporal variability in the yearly time series. Due to the presence of an evident seasonality in the observed mortality rates, a seasonal component was then added to account for time dependence between the same month across different years. Seasonal ARIMA (SARIMA) models were subsequently used to forecast monthly mortality rates for the year 2023, for each scenario and cause of death. The Box and Jenkins [18] approach was employed to fit the parameters of a SARIMA model starting from monthly time series indexed by the time index $k_{t}$. Once the model parameters were estimated by means of a maximum likelihood framework, forecast values were computed, and 95% confidence intervals (CI) were derived under the asymptotic normal approximation for parameter distribution. Results were then presented both as the ratio and as the difference between observed and estimated mortality rates, calculating the relative 95% CI.

Additionally, the Lee‐Carter model was applied as a sensitivity analysis and to explore differences in mortality rates between age groups [19]. This model is one of the most widely used frameworks for mortality forecasting due to its flexibility and relatively simple structure [20]. Let $m_{x,t}$ be the mortality rate for age‐class x at time t. The model applied is:

$$\log(m_{x,t})=a_{x}+b_{x}k_{t}+\xi_{x,t}.$$

Here the $a_{x}$ coefficients describe the average shape of the age profile, and the $b_{x}$ coefficients describe the pattern of deviations from this age profile when time index $k_{t}$ varies. The final term, $\xi_{x,t}$, is the error term, which reflects the age‐specific influences not captured by the model. Mortality forecasting is carried out using the model of the mortality index time series $k_{t}$. An ARIMA model was then used to model the dynamics of $k_{t}$. CIs were constructed by simulating multiple forecast paths for future mortality rates, assuming no parameter uncertainty, accounting for both the forecast variability over time and the fitting error. These simulations were used to generate 95% Pointwise CI.

The analyses were conducted using R statistical software (version 4.1.3), employing Forecast and StMoMo packages. The graphical abstract was prepared with the assistance of NotebookLM (Google).

## 3 Results

**Figure 1:**
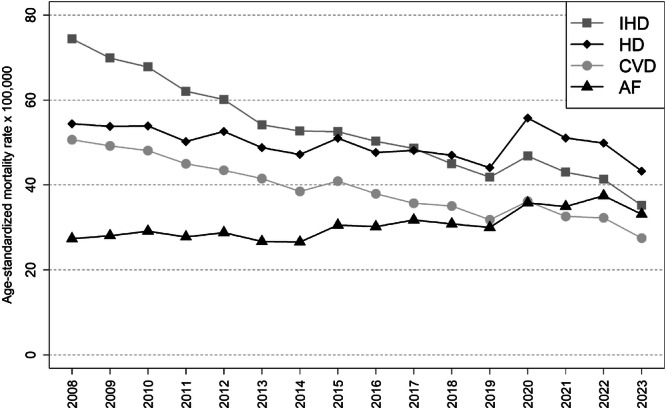
Age‐standardized mortality rates (x100,000; 2013 European standard) for ischemic heart diseases (IHD), cerebrovascular diseases (CVD), hypertensive diseases (HD) and atrial fibrillation (AF). Multiple causes of death, residents in Veneto (Italy) aged ≥ 40 years, 2008–2023.

**Figure 2:**
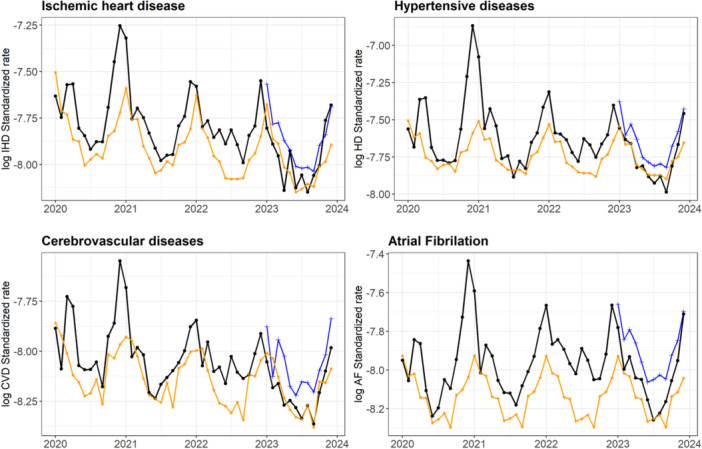
Log‐standardized mortality rates for major circulatory diseases, with forecasts for 2020–2023 based on the 2008–2019 period (orange line with asterisks) and forecasts for 2023 based on the 2008–2022 period (blue line with plus symbols), estimated using SARIMA models.

Overall through the whole study period, 141 072 deaths with any mention of IHD were registered among residents in the Veneto region aged ≥ 40 years; corresponding figures for CVD, HD and AF were 111 978, 145 369 and 88 364, respectively. Distinct trends could be observed before the pandemic: age‐standardized mortality rates were steeply decreasing for CVD and IHD, declining at a slower pace for HD, and increasing for AF. In 2020, a sharp peak could be observed for all the investigated conditions (Figure 1). In 2021–2022 mortality rates were lower than those of the pandemic's first year, but they significantly remained above pre‐pandemic levels. Comparing the observed mortality with that forecasted by 2008–2019 time series, a large mortality excess was registered in 2020, especially for HD (+29%, CI 17%–42%). Such excess mortality persisted through the second and third year of the pandemic, ranging in 2022 from 15% (CVD) to 22% (AF) (Table 1). In the year 2023, the mortality ratio was slightly higher than one, but the unity falls inside the 95% CI for all the diseases considered. Conversely, considering the mortality ratio forecasted on the basis of the 2008–2022 mortality time series, we observed a ratio statistically significantly below 1. Table 2 shows that based on 2008–2019 forecasts, large absolute increases of age‐standardized mortality rates were registered in 2020 (12.5 per 100 000 for HD). By 2023, only minor differences in the observed and forecasted rates based on the 2008–2019 time series were estimated, ranging from 0.7 to 2.4 per 100 000. Conversely, marked negative differences were observed when considering the rate forecasts based on 2018–2022 time series. Figure 2 shows observed and predicted monthly log CVD mortality rates in 2020–2023. Peaks in mortality were registered corresponding to subsequent epidemic waves: involvement of Northern Italy in the early phases of the pandemic in March‐April 2020; the large epidemic wave in November 2020 to January 2021, before vaccine availability; a smaller peak during the third wave in March‐April 2021; the diffusion of the Omicron variant in late 2021 to early 2022. During 2022, increased mortality was fueled both by subsequent COVID‐19 waves and additional factors, including heat waves in July to August in 2022 [21] and the circulation of seasonal respiratory viruses in late 2022. In the year 2023, the observed mortality rates dropped for all the diseases investigated, being close to those predicted based on the 2008–2019 time series. Notwithstanding, in December 2023 an excess mortality could still be observed, in correspondence with the widespread diffusion of multiple respiratory viruses (influenza, respiratory syncytial virus as well as COVID‐19) across Europe [22].

**Table 1:** Ratio between observed and ARIMA forecasted mortality rates, with 95% confidence intervals.

Rate ratio	2020 (A)	2021 (A)	2022 (A)	2023 (A)	2023 (B)
IHD	1.18 (1.11, 1.25)	1.14 (1.05, 1.24)	1.16 (1.04, 1.28)	1.04 (0.92, 1.17)	0.89 (0.80, 0.99)
HD	1.29 (1.17, 1.42)	1.20 (1.04, 1.39)	1.20 (1.01, 1.43)	1.06 (0.87, 1.29)	0.87 (0.74, 1.03)
CVD	1.19 (1.09, 1.29)	1.12 (0.99, 1.26)	1.15 (1.00, 1.32)	1.03 (0.87, 1.21)	0.89 (0.81, 0.99)
AF	1.18 (1.06, 1.33)	1.15 (0.97, 1.35)	1.22 (1.00, 1.49)	1.07 (0.85, 1.34)	0.86 (0.75, 0.99)

*Note:* A: forecast based on 2008–2019 time series; B: forecast based on 2008–2022 time series.

**Table 2:** Difference (per 100.000) between observed and ARIMA forecasted mortality rates, with 95% confidence intervals.

Rate difference per 100.000	2020 (A)	2021 (A)	2022 (A)	2023 (A)	2023 (B)
IHD	7.1 (4.6, 9.4)	5.3 (2.0, 8.4)	5.6 (1.6–9.1)	1.3 (−3.1, 5.1)	−4.4 (−8.8, −0.5)
HD	12.5 (8.2, 16.4)	8.6 (2.0, 14.4)	8.2 (0.3–15.0)	2.4 (−6.5, 9.8)	−6.3 (−15.2, 1.3)
CVD	5.7 (3.0, 8.2)	3.4 (−0.3, 6.7)	4.3 (0–7.9)	0.7 (−4.2, 4.8)	−3.3 (−6.6, −0.4)
AF	5.6 (1.9, 8.9)	4.4 (−1.1, 9.0)	6.7 (0–12.3)	2.1 (−5.9, 8.5)	−5.2 (−10.9, −0.2)

*Note:* A: forecast based on 2008–2019 time series; B: forecast based on 2008–2022 time series.

**Figure 3:**
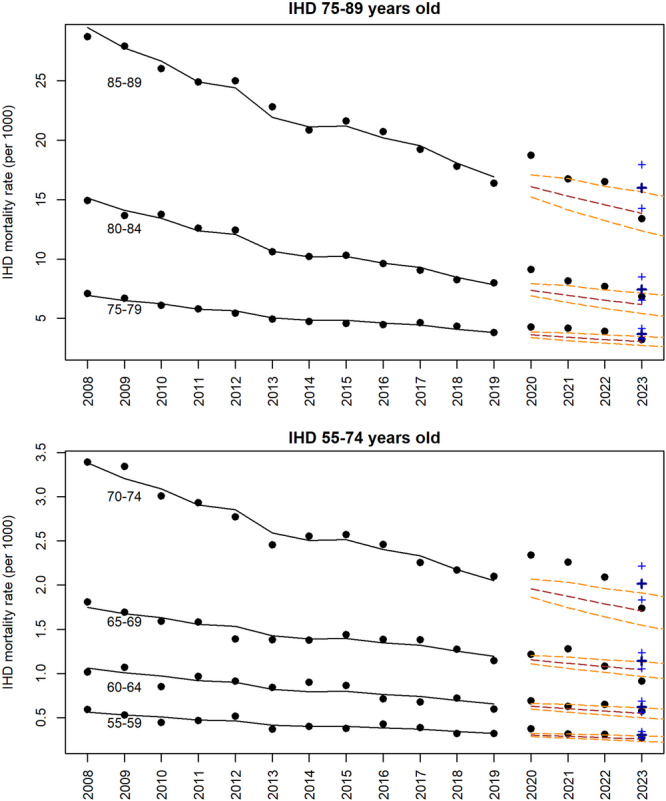
Ischemic heart diseases (IHD) mortality rates (black points), Lee–Carter model fitted values (solid black line), and forecasts for 2020–2023 based on the 2008–2019 time series with 95% pointwise confidence intervals (dashed orange lines), compared with the 2023 point forecast based on the 2008–2022 time series with 95% pointwise confidence intervals (blue plus symbols), by age group.

**Figure 4:**
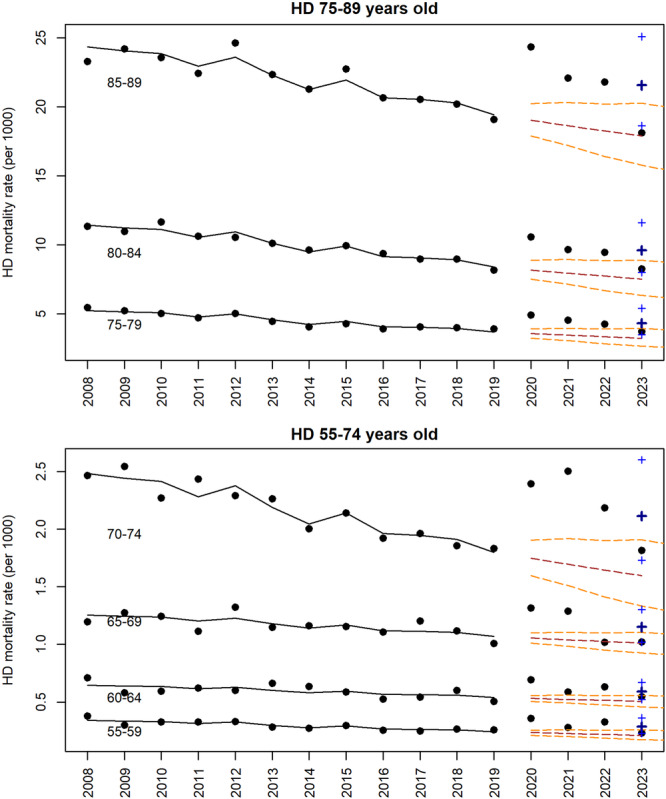
Observed Hypertensive Diseases (HD) mortality rates (black points), Lee–Carter model fitted values (solid black line), and forecasts for 2020–2023 based on the 2008–2019 time series with 95% pointwise confidence intervals (dashed orange lines), compared with the 2023 point forecast based on the 2008–2022 time series with 95% pointwise confidence intervals (blue plus symbols), by age group.

**Figure 5:**
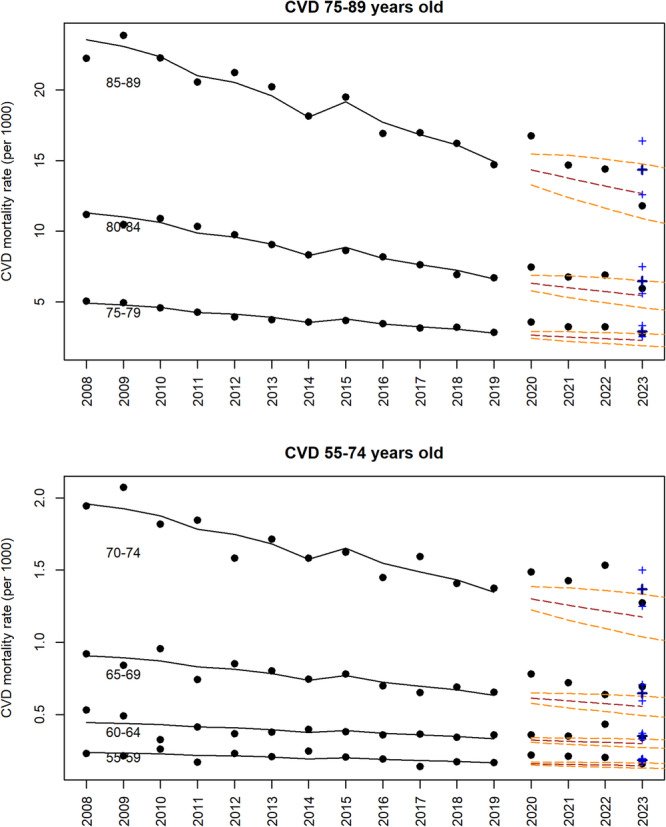
Cerebrovascular diseases (CVD) mortality rates (black points), Lee–Carter model fitted values (solid black line), and forecasts for 2020–2023 based on the 2008–2019 time series with 95% pointwise confidence intervals (dashed orange lines), compared with the 2023 point forecast based on the 2008–2022 time series with 95% pointwise confidence intervals (blue plus symbols), by age group.

**Figure 6:**
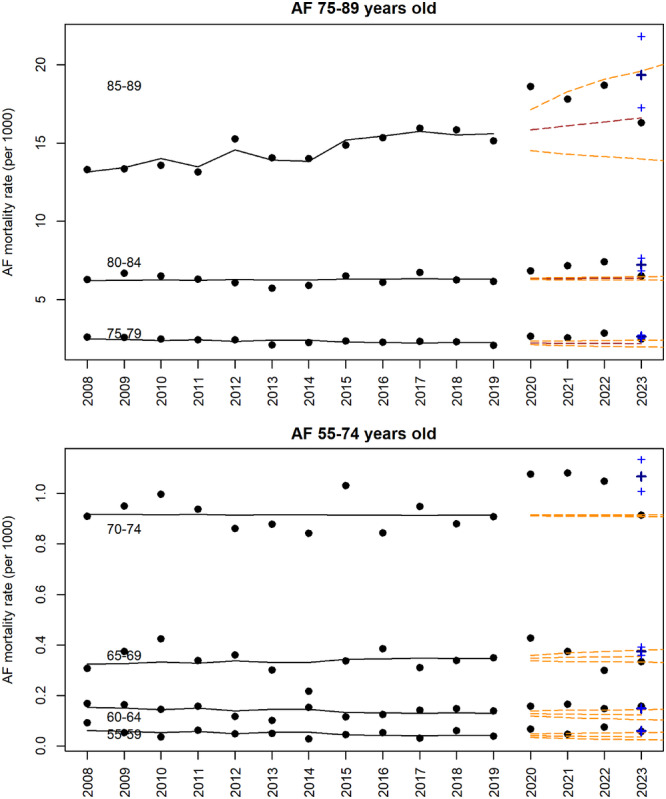
Observed atrial fibrillation (AF) mortality rates (black points), Lee–Carter model fitted values (solid black line), and forecasts for 2020–2023 based on the 2008–2019 time series with 95% pointwise confidence intervals (dashed orange lines), compared with the 2023 point forecast based on the 2008–2022 time series with 95% pointwise confidence intervals (blue plus symbols), by age group.

Figures 3, 4, 5, 6 illustrate results from Lee‐Carter models, confirming that mortality in 2023 tended to re‐align with pre‐pandemic trends. For HD and IHD, mortality rates observed in 2023 fall within the 95% CI of the forecasts based on the 2008–2019 series across all age groups, while forecasts based on the 2008–2022 series consistently lie above the observed rates. Only in some selected age groups for AF and CVD mortality (especially CVD mortality in the 65–69 age class), observed rates fall above the CI of the 2008–2019 forecast and align more closely with the 2008–2022 projections.

## 4 Discussion

Study results show that, in spite of largely diverging pre‐pandemic trends, mortality related to different circulatory diseases followed a common pattern during the pandemic, with a marked increase in 2020–2022 and a return in 2023 towards predicted values based on the 2008–2019 time series. Therefore, the impact of the pandemic was substantial, but its extension beyond 2022 was limited. The present findings also demonstrate that the excess mortality during the whole 2020–2022 pandemic period can be properly estimated accounting for pre‐existing long‐term trends [23]. Notably, in spite of overall rates returning to the baseline, a marked seasonality persisted in 2023, with a mortality peak observed in late 2023, higher than the expected one considering pre‐pandemic trends.

The increase in cardiovascular mortality during the pandemic might be the result of a combination of several direct and indirect effects, acting both in the acute phase and in the long‐term. In fact, older individuals with cardiovascular risk factors and comorbidities such as arterial hypertension, AF/flutter and diabetes mellitus were particularly vulnerable to COVID‐19 adverse outcomes. In the Veneto region, the co‐occurrence of comorbidities, such as diabetes and HD, was associated with a marked mortality increase in 2020–2021 among subjects aged 40–79 years [24]. Furthermore, COVID‐19 is both associated with acute cardiovascular complications, including microvascular thrombosis, and with chronic sequelae with subsequent increased risk of major cardiovascular events [7]. Among indirect effects are reduced and delayed diagnostic and treatment procedures during pandemic waves, due to reorganization of health care with suspension of several medical services, and to reduced access to the hospital for fear of contagion and disruption of social connections. Early studies suggested that hospital admissions for acute cardiac events dropped during lockdowns, leading to worsened outcomes and excess deaths [25]. Decreased physical activity and growing alcohol and tobacco intake might be responsible for increasing cardiovascular risk spanning beyond the pandemic [7]. In spite of all these unfavorable factors, contrasting findings from real‐world data on cardiovascular events have been reported; as an example, in Sweden the incidence of myocardial infarction continued the pre‐pandemic declining trend through 2020–2022, well beyond the initial drop potentially attributable to reduced access to care during the first COVID‐19 waves [26].

While the initial surge in excess deaths globally during the pandemic has subsided, many countries continued to experience higher‐than‐expected overall mortality in 2023, with a high variability across countries [27]. This increase and variability can't be attributed to a single cause, as it's shaped by the complex interplay of diverse national and sub‐national dynamics [28]. For instance, some regions might still be grappling with the lingering health impacts of COVID‐19 infections, disruptions to health care systems, or the exacerbation of chronic conditions [29]. Conversely, other areas might be seeing a rise in non‐COVID‐related deaths, such as those due to drug poisonings, traffic fatalities, or other societal factors, particularly affecting younger adult populations [28].

The available literature does not provide a clear‐cut picture of the long‐term impact of the pandemic on mortality from circulatory diseases. Findings were mostly limited to the US, diverged depending on the disease analyzed, were mainly based on descriptive data from most recent years without taking into account previous trends and seasonality, and were usually limited to the UCOD and therefore greatly influenced by the role of COVID‐19 as a competing cause of death [30]. The present study, based on MCOD, allowed for a more complete estimate of the immediate impact of the pandemic on mortality levels, since it included deaths attributed to COVID‐19 in subjects with chronic cardiovascular conditions [17]. Furthermore, time series analyses of different diseases with diverging pre‐pandemic trends were designed to more properly assess if an impact on cardiovascular mortality persisted after the end of the pandemic. In view of the above, the methodology of the study (MCOD approach, forecasting methods) might be adopted in other geographical areas. The present report addresses a knowledge gap about post‐pandemic cardiovascular mortality trends outside the US; although findings might be generalized to other European populations, further studies from other countries and extended to more recent years are warranted. In fact, data were limited to 2023, and study results must be confirmed by analyses involving a longer post‐pandemic period. Nonetheless, the finding that in 2023 cardiovascular mortality figures in the Veneto Region substantially realigned to pre‐pandemic trends is of interest for both policymakers and the general public, both concerned about a major long‐term impact of the pandemic on population‐level mortality rates.

A limitation of this analysis is that the forecasted mortality rates were estimated under the assumption of no parameter uncertainty. This may result in an underestimation of the true variability of the projections. Future studies could evaluate the effect of including parameter uncertainty under different approaches in order to provide more robust and reliable interval estimates.

Although the present results suggest an overall favorable scenario, with mortality related to common circulatory diseases after the end of the pandemic returning to trends observed in 2008–2019, in the case of AF this means re‐aligning to a previous long‐term increase. A growth in AF‐related mortality through the last decades has been reported also in the US, possibly due to the ageing of the population and diffusion of risk factors such as diabetes mellitus and obesity [31]. Therefore, continuous monitoring of mortality data is warranted to assess if all the observed tendencies will continue through the next post‐pandemic years.

## Author Contributions

All authors contributed to the study conception and design. Data collection was performed by Ugo Fedeli and analyses were performed by Giulia Capodaglio. The first draft of the manuscript was written by Giulia Capodaglio and Ugo Fedeli; all authors commented on previous versions of the manuscript. All authors read and approved the final manuscript.

## Funding

The authors have nothing to report.

## Ethics Statement

This is an observational study carried out on anonymous mortality records. The analysis of mortality data is a mandatory activity of the Regional Epidemiological Department according to the regional law. Therefore, this study was exempt from Institutional Review Board approval.

## Conflicts of Interest

The authors declare no conflicts of interest.

## Data Availability

The data that support the findings of this study are available from the corresponding author upon reasonable request.
